# Tracheobronchial tooth and dental prosthesis aspirations: 15 cases

**DOI:** 10.1186/s13019-023-02178-5

**Published:** 2023-02-21

**Authors:** Aykut Eliçora, Hüseyin Fatih Sezer, Salih Topçu, Tülay Çardaközü

**Affiliations:** 1 Department of Thoracic Surgery, Kocaeli University Medical Faculty, Izmit, Kocaeli Turkey; 2 Department of Thoracic Surgery, Kocaeli University Medical Faculty, Izmit, Kocaeli Turkey; 3 Department of Thoracic Surgery, Kocaeli University Medical Faculty, Izmit, Kocaeli Turkey; 4 Department of Anesthesiology and Reanimation, Kocaeli University Medical Faculty, Izmit, Kocaeli Turkey

**Keywords:** Aspiration, Dental prosthesis, Foreign body, Tooth

## Abstract

**Background:**

Tracheobronchial foreign body is uncommon in adults. Among foreign body aspirations, tooth and dental prosthesis aspiration is a very rare condition. In the literature, dental aspiration is generally found as a case report and there is no single-center case series. In this study, we aimed to present our clinical experience in 15 cases with tooth and dental prosthesis aspiration.

**Methods:**

Data from 693 patients who presented to our hospital for foreign body aspiration between the years 2006 and 2022 were analyzed retrospectively. Fifteen cases who aspirated tooth and dental prostheses as foreign bodies were included in our study.

**Results:**

Foreign bodies were removed by rigid bronchoscopy in 12 (80%) cases and fiberoptic bronchoscopy in 2 (13.3%) cases. In one of our cases, foreign body was expected with cough.When evaluated in terms of foreign body, partial upper anterior tooth prosthesis in 5 (33.3%) cases, partial anterior lower tooth prosthesis in 2 (13.3%) cases, dental implant screw in 2 (13.3%) cases, lower molar crown in 1 (6.6%) case, lower jaw bridge prosthesis in 1(6.6%) case, upper jaw bridge prosthesis in 1(6.6%) case, broken tooth fragment in 1(6.6%) case, upper molar tooth crown coating in 1(6.6%) case and upper lateral incisor tooth in 1(6.6%) case were observed.

**Conclusion:**

Dental aspirations can also occur in healthy adults. Anamnesis is the most important factor in diagnosis and diagnostic bronchoscopic procedures should be performed in cases where adequate anamnesis cannot be obtained.

## Introductıon

Tracheobronchial foreign body aspiration is one of the life-threatening emergencies. It is most common in children aged 1–3 years. Tracheobronchial foreign body aspirations are very rare in adults. However, it has been reported that there is a significant increase in the frequency of foreign body aspiration in adults after the age of fifty years [1]. Tracheobronchial foreign body aspirations can be asymptomatic or cause serious respiratory complications. In some cases, it can be mortal. Aspiration of tooth and dental prostheses as a foreign body is mostly observed as a result of maxillofacial injuries or intraoral interventions [2]. In addition, tooth and dental prosthesis aspirations can be seen more frequently in the elderly, alcoholics, motor-mental retardation or neurological diseases. Dental prosthesis aspirations have a rate of 0.4% among all foreign body aspirations and are seen very rarely [3]. Tooth and dental prosthesis aspirations are frequently found as case reports in the literature, and there is no single-center case series in the literature. In this study, we aimed to present our clinical experience in 15 cases with tooth and dental prosthesis aspiration.

## Materıal and method

Data from 693 patients who presented to our hospital for foreign body aspiration between the years 2006 and 2022 were analyzed retrospectively. Fifteen cases who aspirated tooth and dental prostheses as foreign bodies were included in our study. The cases were evaluated in terms of age, gender, physical examination, medical history, duration of dental prosthesis, symptoms, admission time to the hospital, radiological examination and radiological findings, nature and localization of foreign body, type of anesthesia, bronchoscopic procedure type and complications. Chest X-rays and thorax computerized tomography (CT-scans) were used in the radiological evaluation of the cases. Hemogram, coagulation factors and routine biochemistry tests were performed in all cases.

A rigid and flexible bronchoscope was used to remove foreign bodies. Oral feeding was stopped 6–8 h before all bronchoscopic procedures. All bronchoscopic procedures were performed under operating room conditions. Aerosol lidocaine (max 8.2 mg/kg) as a local anesthetic and intravenous midazolam (0.06–0.07 m/kg) for sedation were administered before a flexible bronchoscopy procedure (Karl Storz Instruments, Germany). Rigid bronchoscopy (Karl Storz Instruments, Germany) procedures were performed under general anesthesia. In rigid bronchoscopy, foreign bodies were removed using crocodile and peanuts forceps. Foreign bodies were removed using basket forceps in flexible bronchoscopy.

Statistical analysis was done with IBM SPSS 20.0 (IBM Corp., Armonk, NY, USA) package program. Normal distribution was evaluated with the Shapiro–Wilk test. Numerical variables were given as mean ± standard deviation. Categorical variables were given as frequency (percentage). Comparison of continuous variables between groups was carried out using Mann–Whitney U test. All statistical analyses were carried out with 5% significance and a two-sided *p*-value < 0.05 was considered as statistically significant.

## Results

A total number of 15 cases were included in our study. In terms of gender distribution, 5 (33.3%) of our cases were female and 10 (66,7%) were male. The mean age of the cases was 58.10 ± 9.07 (range of 32 to 71) years. The mean age was 63.43 ± 10.13 (range of 47 to 82) years in males and 47.00 ± 10.36 (range of 32 to 59) years in females. No statistically significant difference was found between the male and female groups in terms of age distribution (*p* = 0.06).

In the medical history of the cases, 6 patients (40%) had hypertension, 6 (40%) had coronary artery disease, 5 (33.3%) had diabetes mellitus, 4 (26.6%) had obstructive sleep apnea syndrome, 4 (26.6%) had chronic obstructive pulmonary disease, 4 (26.6%) had bruxism, 3 (20%) had asthma, 3 (20%) had depression, 3 (20%) had alcoholism and 1 (6.6%) had myasthenia graves (Table 1).

**Table 1:** Medical history of patients

History	Number of patients (%)
Hypertension	6 (40)
Coronary artery disease	6 (40)
Diabetes mellitus	5 (33.3)
Obstructive sleep apnea syndrome	4 (26.6)
Chronic obstructive pulmonary disease	4 (26.6)
Bruxism	4 (26.6)
Asthma	3 (20)
Depression	3 (20)
Alcoholism	3 (20)
Myasthenia graves	1 (6,6)

The most common symptoms were cough and dyspnea. Hemoptysis was observed in two cases. On physical examination, wheezing in 11 (73.3%) cases, decrease in breathing sounds in 9 (60%) cases, stridor in 6 (40%) cases, decreased saturation in 4 (26.6%) cases and there was no finding in 4 (26.6%) cases (Table 2).

**Table 2:** Physical examination signs and symptoms

Physical examination	Number of patients (%)	Symptom	Number of patients (%)
Wheezing	11 (73.3)	Cough	10 (66.6)
Decrease in breathing sounds	9 (60)	Dyspnea	9 (60)
Stridor	6 (40)	Hemoptysis	2 (13.3)
Decreased saturation	4 (26.6)	Asymptomatic	6 (40)
High mechanical ventilator pressure	1 (6.6)		
No finding	4 (26.6)		

Thirteen of our cases were admitted to the hospital within twenty four hours after foreign body aspiration. The mean time of admission to the hospital after foreign body aspiration was found to be 14.21 ± 5.28 (range 2 to 24) hours. The diagnosis of foreign body was made three weeks later in one patient who was in the intensive care unit and aspirated his tooth after trauma. A case who aspirated the molar tooth crown did not remember the time of aspiration.

A chest X-ray was performed for radiological examination in all cases. Computerized tomography of thorax was performed in 9 (60%) cases. On radiological evaluation, all of the foreign bodies were detected directly.

**Fig. 1:**
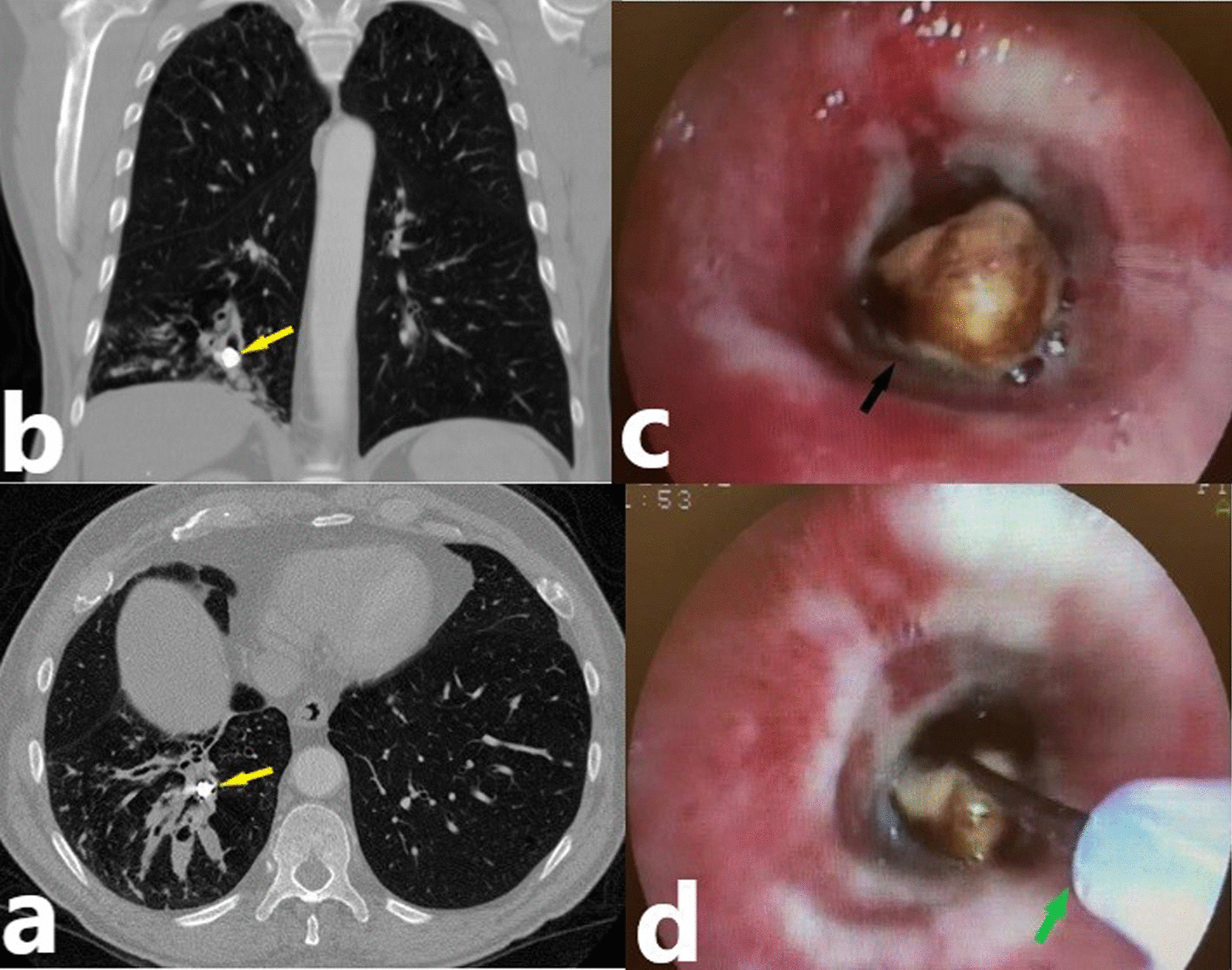
Removal of the lower molar crown with a fiberoptic bronchoscope. **a**,** b** Axial and coronal sections of computed tomography of the thorax; yellow arrow shows molar crown in the right lower lobe bronchus.** c** Foreign body appearance on fiberoptic bronchoscopy; black arrow shows molar crown. **d** Removal of the foreign body with basket forceps

**Fig. 2:**
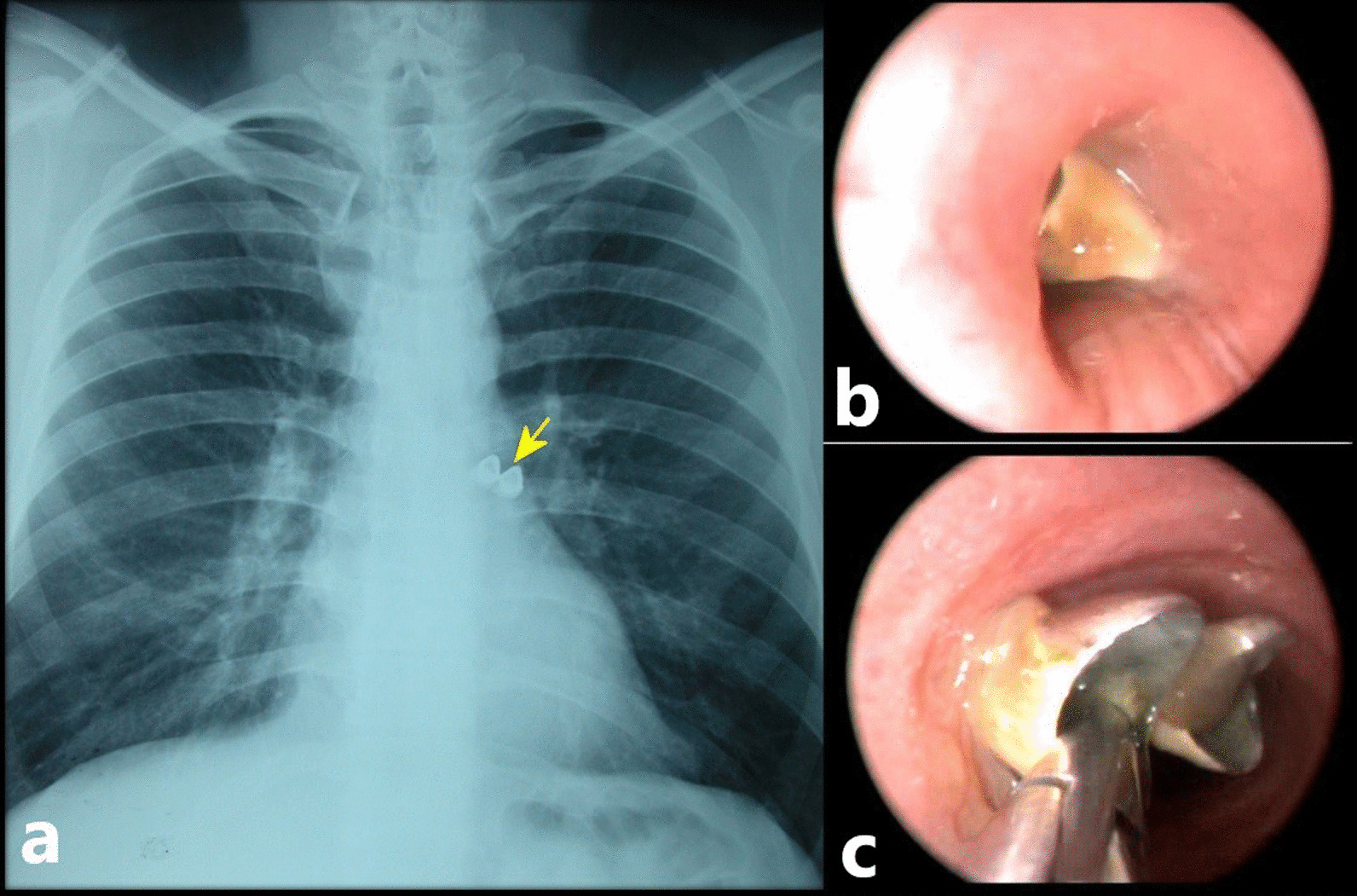
Removal of partial upper anterior tooth prosthesis with rigid bronchoscope**. a** Posterior-Anterior (PA) chest X-ray; black arrow shows foreign body in the left main bronchus. **b**, **c** Rigid bronchoscopy view of upper anterior tooth prosthesis

**Fig. 3:**
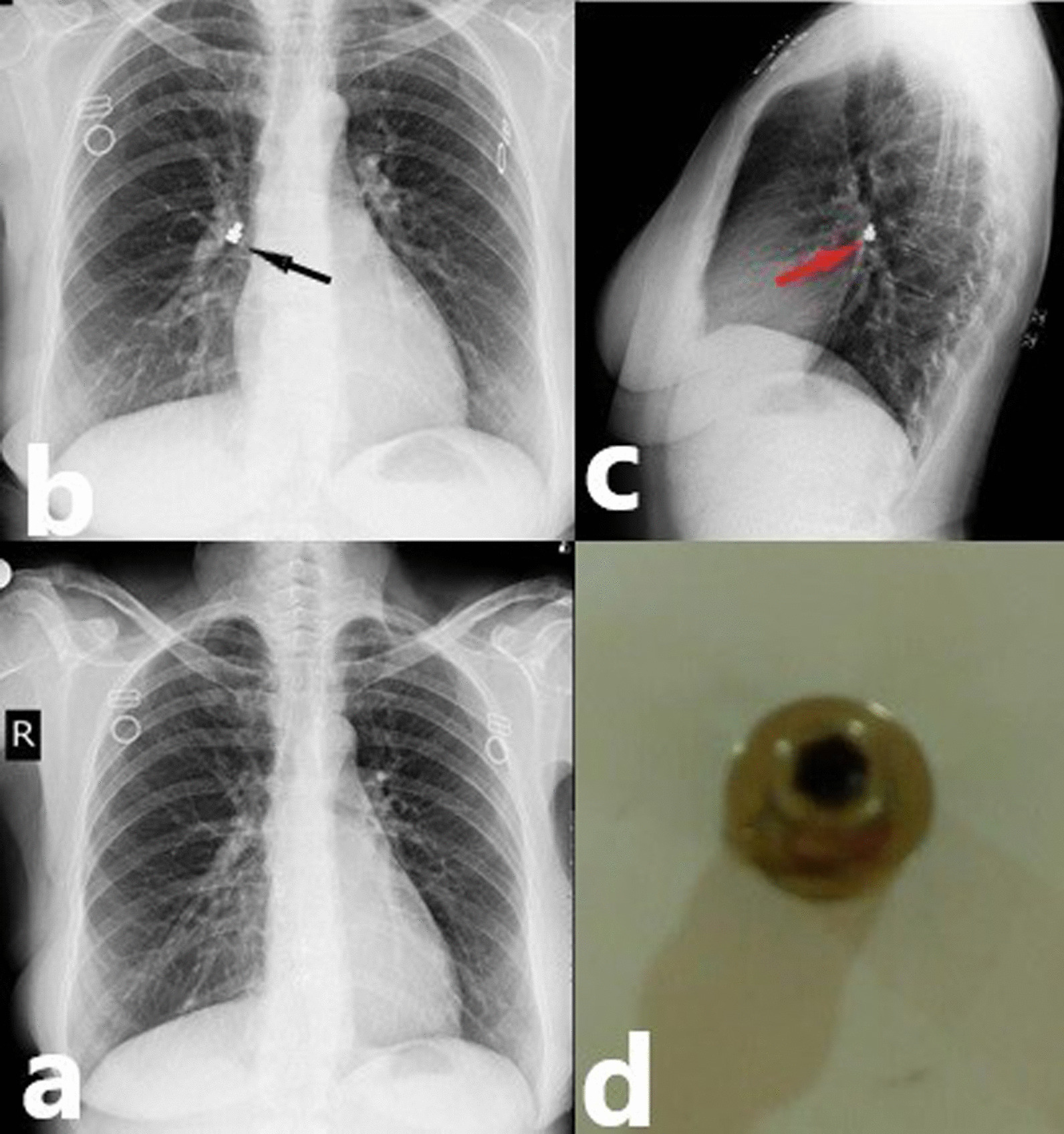
Dental screw expectorated by cough. **a** Posterior-Anterior (PA) chest X-ray; after expectorated. **b**,** c** Posterior-Anterior (PA) and lateral chest X-ray; black and red arrow shows dental screw. **d** Dental screw

Foreign bodies were removed by rigid bronchoscopy in 12 (80%) cases and fiberoptic bronchoscopy in 2 (13.3%) cases ( Fig. 1). General anesthesia was applied in 11 (73.3%) patients who underwent rigid bronchoscopy (Fig. 2), while local anesthesia was applied in 2 (13.3) patients who underwent fiberoptic bronchoscopy. No anaesthesia was used in one (6.6%) patients who required bronchoscopy under intensive care unit conditions. In one of our cases, foreign body was expected with cough (Fig. 3).

**Fig. 4:**
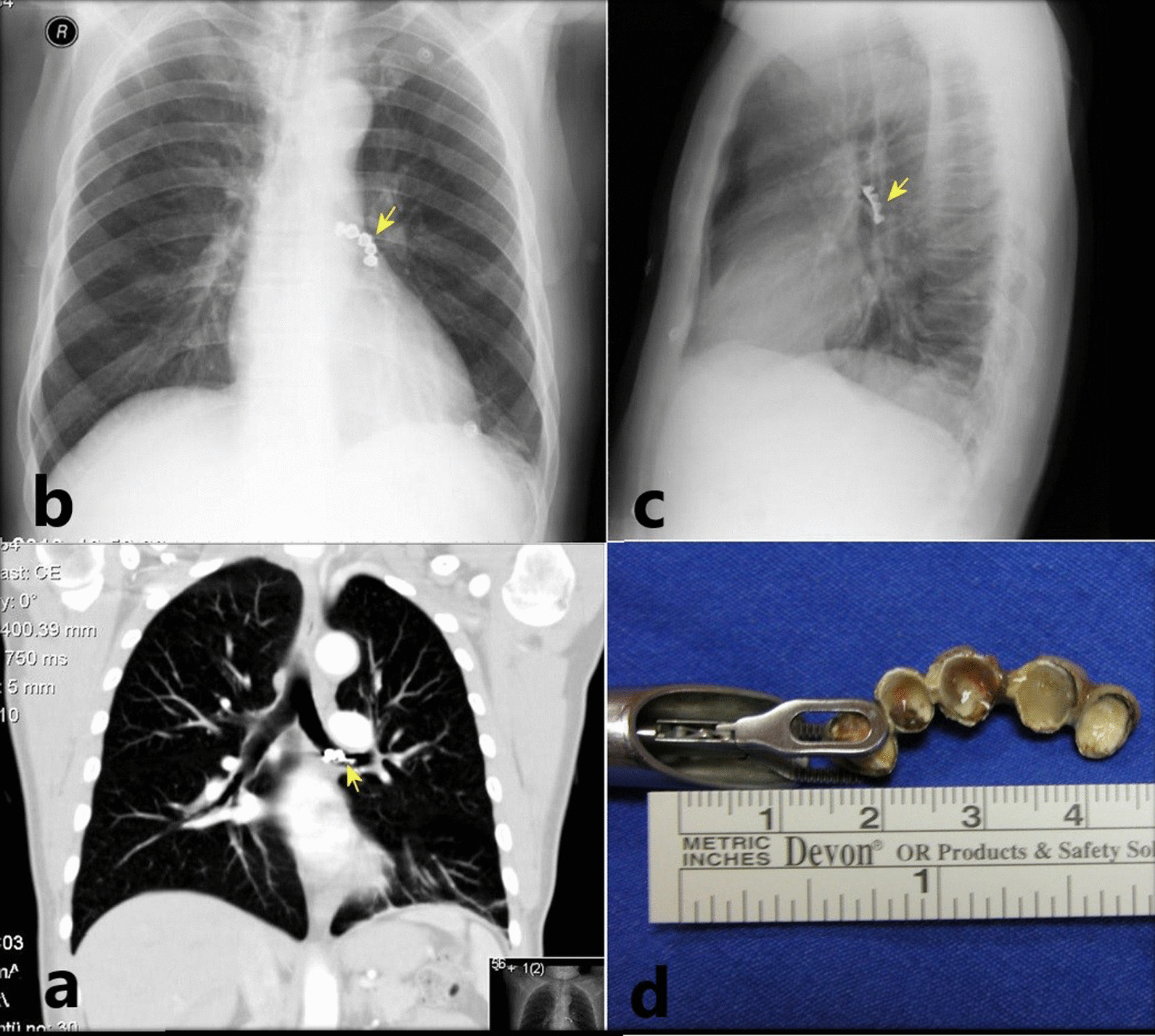
Lower jaw bridge prosthesis. **a** Coronal section of computed tomography of the thorax; yellow arrow shows molar crown in the left main lobe bronchus. **b**,** c** Posterior-Anterior (PA) and lateral chest X-ray; black and red arrow shows dental screw. **d** Rigid bronchoscope forceps and bridge prosthesis

When evaluated in terms of foreign body, partial upper anterior tooth prosthesis in 5 (33.3%) cases (Fig. 2), partial anterior lower tooth prosthesis in 2 (13.3%) cases, dental implant screw in 2 (13.3%) cases, lower molar crown in 1 (6.6%) case, lower jaw bridge prosthesis in 1(6.6%) case (Fig. 4), upper jaw bridge prosthesis in 1(6.6%) case, broken tooth fragment in 1(6.6%) case, upper molar tooth crown coating in 1(6.6%) case and upper lateral incisor tooth in 1(6.6%) case were observed (Table 3). All of our 7 (46,6%) cases who aspirated partial tooth prosthesis had total tooth prosthesis. The mean duration of the prosthesis was found to be 8.90 ± 4.01 (range of 2 to 14) years in cases who aspirated the prosthesis.

**Table 3:** General characteristics

Age	Gender	Foreign body	Time of dental prosthesis (year)	Localization	Method of removal
32	Female	Partial upper anterior tooth prosthesis	2	Right main bronchus	Rigid bronchoscopy
42	Male	Partial upper anterior tooth prosthesis	6	Left main bronchus	Rigid bronchoscopy
50	Female	Dental implant screw	–	Right main bronchus	Rigid bronchoscopy
57	Male	Partial anterior lower tooth prosthesis	11	Left main bronchus	Rigid bronchoscopy
67	Male	Lower molar crown	–	Right lower lobe bronchus	Fiberoptic bronchoscopy
71	Male	Lower jaw bridge prosthesis	14	Left main bronchus	Rigid bronchoscopy
53	Female	Partial upper anterior tooth prosthesis	7	Right main bronchus	Rigid bronchoscopy
63	Male	Partial upper anterior tooth prosthesis	12	Right main bronchus	Rigid bronchoscopy
68	Male	Upper jaw bridge prosthesis	13	Right main bronchus	Rigid bronchoscopy
50	Male	Partial upper anterior tooth prosthesis	4	Right main bronchus	Rigid bronchoscopy
54	Male	Broken tooth fragment	–	Right lower bronchus	Fiberoptic bronchoscopy
43	Female	Dental implant screw	–	Right main bronchus	Expected with cough
59	Female	Partial anterior lower tooth prosthesis	11	Left main bronchus	Rigid bronchoscopy
56	Male	Upper molar tooth crown	9	Right main bronchus	Rigid bronchoscopy
53	Male	Upper lateral incisor tooth	–	Right main bronchus	Rigid bronchoscopy

Foreign bodies were evaluated in terms of location as follows: 9 foreign bodies (60%) were in the right main bronchus, 4 (26.6%) in the left main bronchus and 2 (13.3) in the right lower lobe bronchus.

As a complication, mucosal injury in the trachea during the removal of the foreign body in 3 (20%) cases and hoarseness due to edema in the vocal cords in 2 (13.3%) cases occurred. In the controls performed one week after bronchoscopy, no finding was found in the physical examination of the cases.

In our study, the rate of tooth and dental prosthesis aspiration was found to be 2.2%.

## Dıscussıon

Tracheobronchial foreign body aspirations are less common than gastrointestinal foreign bodies. However, foreign bodies in the tracheobronchial system cause higher mortality and morbidity than the gastrointestinal system. While the mortality rate in tracheobronchial system foreign bodies is approximately 5–7%, this rate is around 0.85% in gastrointestinal tract foreign bodies [4, 5]. Aspirated foreign bodies can be localized in any area from the larynx to the distal bronchioles. The localization of the foreign body in the airway varies according to the position of the patient and the size of the object. Aspirated foreign bodies are often localized to the right main bronchus because the right main bronchus is wider and more vertical than the left main bronchus [6, 7]. The type of aspirated foreign body varies according to the patient's age, gender, beliefs, socio-economic status and nutritional characteristics. [6]

Foreign body aspirations are evaluated in terms of gender, it is more common in men [8]. The majority of our cases consisted of male patients. While organic foreign body aspirations are more common in the pediatric age group, inorganic foreign body aspirations are more common in adults. Tooth and dental prosthesis aspirations in adults are a very rare type of aspiration. In our study, the rate of tooth and dental prosthesis aspiration was found to be 2.2% when compared with all foreign bodies.

Dental caries occurs in elderly patients due to deterioration in oral hygiene. Although a decrease in the prevalence of tooth decay has been observed worldwide, untreated tooth decay in permanent tooth is quite common and occurs in approximately 35% of the world's population [9]. Tooth decay can cause the crowns on the tooth to break or loosen. In a study of ceramic-based dental prostheses applied due to tooth loss, more than 85% of fixed dental prostheses can remain functional for five years [9, 10]. The functional duration time of the prosthesis is affected by many factors, especially the number of tooth, the position of the tooth in the arch, periodontal health, tooth structure, cement type, and the skill of the dentist [9]. Although dental prostheses such as bridges are more conservative and appropriate, they are used less frequently due to fear of separation. In our cases, aspiration of metal coated ceramic bridge prostheses was more common. The most frequently aspirated foreign bodies in our study were prostheses of the anterior tooth and prostheses used for more than five years. In the literature, loose adhesion of dental prostheses and use of incomplete cement have been defined as factors related to easy detachment of the prosthesis.

Maxillofacial trauma and emergency intubations are among the predisposing factors in dental aspirations [11, 12]. For this reason, it should be checked whether there are missing tooth in the mouth after maxillofacial trauma. Likewise, in cases where intubation is performed under emergency conditions, the oral cavity must be checked. In such cases, the history cannot be taken because the patients are unconscious, and therefore the diagnosis of foreign body may be delayed [6]. In our case, whose tooth aspirated due to trauma, the diagnosis was made incidentally in the third week of hospitalization. For this reason, evaluation with thorax computer tomography is very helpful in early diagnosis in cases intubated due to trauma.

During dental treatments, some dental tools or screws can be aspirated. In our two cases, the dental screw was aspirated. Therefore, some preventive measures have been recommended to dentists. These include the use of an intraoral protective cover, adequate secretion cleaning so that patients do not need to breathe deeply, and tying some prosthesis parts with a thread [13]. However, in practice, it has been reported that the rate of implementation of these measures by dentists is below 20%. [13]

Detailed anamnesis is very important in the diagnosis of all foreign body aspirations. Sudden onset of cough and respiratory distress in the patient's history is diagnostic for foreign body aspiration. The diagnosis of foreign body aspiration may be delayed in cases where adequate anamnesis cannot be obtained. Delayed diagnosis in foreign body aspirations may cause recurrent lung infections, lung abscesses, bronchiectasis, hemoptysis, and formation of granulation tissues that can be confused with lung cancer [14]. Bronchiectasis developed due to recurrent infections in one of our cases who aspirated the molar tooth crown. This case did not remember the time of aspiration. Foreign body aspiration should be excluded in cases where frequent pneumonia develops or airway pressure is high in cases followed in the intensive care unit due to trauma. After the anamnesis, radiological examinations are required to confirm the diagnosis. Chest X-ray is the most commonly used radiological examination. Radiopaque foreign bodies can be easily seen on chest X-ray. However, non-radiopaque or semiopaque objects such as tooth may not be directly visible on chest radiographs. Non-opaque foreign bodies may cause secondary radiological findings such as increased aeration on the ipsilateral lung or atelectasis. In order to determine the localization of the foreign body correctly, it should be evaluated with posteroanterior and lateral chest radiographs. In our study, a patient who aspirated an implant screw had expected a foreign body with cough before bronchoscopy. Therefore, repeating chest X-ray before bronchoscopy in foreign body aspirations gives information about whether the localization of the foreign body has changed. Computed tomography of the thorax is a very important radiological examination to confirm the foreign body. However, its routine use in foreign body aspirations is limited. It is frequently used diagnostically in patients with foreign body clinical findings and normal chest X-ray. It should be kept in mind that recurrent lung infections may develop in foreign body aspirations and should be evaluated with thorax computed tomography.

Bronchoscopic methods are most commonly used in the treatment of foreign body aspirations. Although the most commonly used method is rigid bronchoscopy, fiberoptic bronchoscopy can be used in the treatment of foreign body aspiration. The advantages of fiberoptic bronchoscopy are that it can be performed under local anesthesia and sedation. Disadvantages of fiberoptic bronchoscopy are that the forceps are quite small, there is a risk of foreign body escaping back into the airway, and small foreign bodies can be removed [14]. In our study, foreign bodies of two cases were removed with a fiberoptic bronchoscope. Rigid bronchoscopy is a procedure performed under general anesthesia, and foreign bodies can be removed and complications such as hemoptysis can be treated. Since the edges of metal-coated dental prostheses are very sharp, they can damage the tracheal wall and vocal cords during removal. It has been reported that s.epidermidis sepsis may occur due to damage to the mucosa during the removal of dental prostheses [15]. For this reason, the largest diameter rigid bronchoscope should be used in the removal of dental prostheses and the foreign body should be kept inside the rigid bronchoscope as much as possible. In our study, tracheal mucosa was injured in three cases, but s.epidermidis infection was not observed. Foreign bodies that cannot be removed by bronchoscopic methods should be removed by thoracotomy.

In conclusion, tracheobronchial aspiration of tooth and dental prostheses is quite rare. Although foreign body aspirations are frequently seen in adults with advanced age, dementia, alcoholism, neurological diseases and maxillofacial trauma, it should be kept in mind that dental aspirations can also occur in healthy adults. Anamnesis is the most important factor in diagnosis, and diagnostic bronchoscopic procedures should be performed in cases where adequate anamnesis cannot be obtained.

## Data Availability

Kocaeli University Nucleus System. Manuscript has not been presented in a congress or meeting.
